# Psychometric Properties of the Bangla Version of Weinstein Noise Sensitivity Scale Short Form (NSS‐SF‐BV) Among Bangladeshi Individuals: A Cross‐Sectional Study

**DOI:** 10.1002/hsr2.70884

**Published:** 2025-05-26

**Authors:** Pramath Chandra Sarker, Md. Nur‐E‐Alam Siddique, Sabina Sultana

**Affiliations:** ^1^ Department of Psychology Rajshahi Government College Rajshahi Bangladesh; ^2^ Institute of Environmental Science University of Rajshahi Rajshahi Bangladesh; ^3^ Department of Psychology University of Rajshahi Rajshahi Bangladesh

**Keywords:** adaptation, noise, noise pollution, noise sensitivity, psychometric properties

## Abstract

**Background and Aims:**

The Weinstein Noise Sensitivity Scale (NSS) was developed to measure the noise sensitivity of the students. However, until now, NSS tools have not been adapted or assessed for psychometric properties in Bangla. The present study aims to investigate the psychometric properties of the Bangla Version of the Noise Sensitivity Scale Short Form (NSS‐SF‐BV) in both student and general populations.

**Methods:**

A cross‐sectional survey was conducted with convenience sampling among the participants. A total of 793 participants completed the NSS‐SF‐BV, Noise Annoyance Scale (NAS) and Depression, Anxiety and Stress Scale (DASS‐21) questionnaire in this study. The student sample consisted of 491 students aged 18–28 (M = 22.90, 41.8% males), and the general sample consisted of 302 adults aged 19–69 (M = 36.90, 50.3% males) from Rajshahi city, Bangladesh. The factor structure was tested using exploratory factor analysis (EFA) and confirmatory factor analysis (CFA) with two independent samples. 81 participants completed the NSS‐SF‐BV twice over a 2‐week interval to ensure test‐retest reliability.

**Results:**

The EFA confirmed the one‐factor structure of the NSS‐SF with five items, explained 52.23% of the total variance in the student sample. The CFA confirmed the unidimensional factor and approved the structural validity of the scale. Internal consistency as measured by Cronbach's alpha and Omega demonstrated satisfactory reliability (student sample: *α* = 0.76, *ω* = 0.79; general sample: *α* = 0.75, *ω* = 0.79). Concurrent and convergent validity (student sample 0.86 and general sample 0.71) were found satisfactory. The model fit indices were acceptable. Positive and strong correlations were observed between the NSS‐SF‐BV and NAS (r = 0.381, *p* = 0.01), along with a significant, although weak correlation with stress (*r* = 0.089, *p* = 0.05).

**Conclusion:**

Considering all the results, the NSS‐SF‐BV is a valid and reliable tool for evaluating people's noise sensitivity in Bangladesh.

## 1 Introduction

Noise pollution, or unwanted sound, is a global health issue. It was ranked as the leading environmental stressor by the World Health Organization [1]. This phenomenon occurs frequently in Bangladesh and other countries. Extensive urbanization and development have commenced, and Bangladesh remains a nation with a high population density [2]. Development has escalated noise pollution above permissible thresholds. Many health issues are linked to noise sensitivity and noise annoyance [3]. Studies have found a link between noise sensitivity and several physical and mental health issues, including a higher risk of heart disease [4, 5], sleep problems [6, 7], cognitive function [7], psychological well‐being [8], and quality of life [7]. Noise sensitivity is linked to hypertension, cognitive functions and other factors [9, 10, 11].

Although noise exposure, or noise level, is an objective measure, noise sensitivity is a subjective experience. Individuals perceive and react to the same sound level differently, leading to diverse levels of noise sensitivity. Individuals with noise sensitivity respond more rapidly and acutely to auditory stimuli than their counterparts. Conversely, noise‐sensitive individuals may adapt with greater difficulty and at a somewhat slower pace. Furthermore, individuals who are acutely sensitive to noise and cannot disregard it encounter significant challenges in their daily lives. Their mental health deteriorates significantly as a consequence [12].

In a longitudinal study, Weinstein [13] developed the Noise Sensitivity Scale (WNSS‐21), a prominent unidimensional self‐reported instrument designed to assess the noise sensitivity of college dormitory students. The scale evaluates affective responses and attitudes towards general noise pollution and everyday environmental noise. The WNSS has been translated or validated in numerous languages, including Swedish [14], German [15], Serbian [16], Italian [17], Russian [18], Persian [19], Arabic [20], Japanese [21], Chinese [22, 23], and Turkish [24]; however, it has not been translated, validated or evaluated for psychometric properties in the Bengali language.

The original scale had 21 items. It was sometimes too long for time‐sensitive field settings. Numerous researchers attempted to shorten and enhance the scales' practicality for field use. The authors identified two short forms of WNSS (e.g., the NSS 10‐item scale [21] and the 5‐item scale [23, 25, 26]). Following item reduction, the NSS 5‐item scale remains unidimensional, like the WNSS, and exhibits excellent psychometric properties and internal consistency. The five‐item NSS‐SF demonstrated internal reliability, temporal stability, and a significant correlation with the long scale. The Cronbach alpha estimates, ranging from 0.70 [12], to 0.80, [14, 15] and alongside robust test‐retest reliability, were reported by Zimmer & Ellermeier [15] at 0.87 over a 4‐week interval, Weinstein [13] at 0.75 over a 9‐week interval, and Stansfeld [12] at 0.75 over a 4‐month interval (Table 1). Researchers have reported this scale's reliability and validity of this measure among adult samples, laboratory settings, academic settings, parks, and several other settings [25]. However, this scale has several limitations and criticisms [21, 29].

**Table 1:** Various cross‐cultural adaptations of NSS.

Author(s)	Version and items	Sample size	Internal consistency	Validity	Model fits indices/factor loadings
Azzi et al. [20]	Arabic version/4 items	527 Lebanese adolescents	*α* = 0.84, *ω* = 0.84	AVE = 0.74, CV = 0.32	*χ* ^2^ = 5.07, *df* = 4, *p* < 0.001, CFI = 0.992, TLI = 0.976, RMSEA = 0.076, FL = 0.65 – 0.81
Benfield et al. [25]	English version/21 and 5 items	A total of five samples	*α* = 0.73 to 0.83, test‐retest = 0.83	—	CFI = 0.97, RMSEA = 0.078, FL = 0.564–0.701
Dzhambov and Dimitrova [26]	Bulgarian version/5 items	n1 = 115, n2 = 71	*ω* = 0.923, test‐retest, *r* = 0.99	AVE = 0.906, Discriminant = 0.718	*χ* ^2^ = 7.55, *df* = 5, *p* = 0.183, CFI = 0.996, RMSEA = 0.067, SRMR = 0.011, FL = 0.88–0.94
Zhong et al. [23]	Chinese version/5 items	n1 = 187, n2 = 186	*α* = 0.715 and 0.712, CR = 0.720 and .726	Nomological validity = 0.155	*χ* ^2^ = 9.207, *df* = 5, *p* = 0.101, CFI = 0.972, TLI = 0.944, SRMRFL = Factor loading = 0.037, FL = 0.53 – 0.65;
Miller et al [27]	Chinese version/5 items	n1 = 323, n2 = 397	*α* = 0.73 and 0.77	—	*χ* ^2^ = 7.34, *df* = 5, *p* = 0.119, CFI = 0.994, TLI = 0.985, RMSEA = 0.046, FL = 0.32 – 0.85;
Senese et al. [17]	Italian version/20 items	413 adults	*α* = 0.863, split‐half = 0.849	Nomological validity = 0.237	*χ* ^2^ = 609.3, *p* < 0.001, CFI = 0.93, RMSEA = 0.079, FL = 0.33–0.77
Zimmer and Ellermeier [15]	German version	213 students	*α* = 0.70 − 0.92, Test‐retest *r* = 0.87	Divergent validity = 0.165‐.247	—
Ekehmmar & Dornic [14]	Swedish version	236 students	*α* = 0.84	CV = −0.334	—
Weinstein [13]	English version/21 items	—	*α* = 0.83, test‐retest = 0.75	—	—
Yildiz et al. [24]	Turkish version/21 items	—	Test‐retest = 0.92	—	*χ* ^2^ = 267.62, *df* = 5, *p* = 0, CFI = 0.888, TLI = 0.872, RMSEA = 0.053, FL = 0.33 – 0.83
Li et al. [28]	Chinese version	1069 Chinese adults	*α* = 0.72‐.83	AVE = 0.272 and 0.268	*χ* ^2^ = 40.01, CFI = 0.923, RMSEA = 0.073
Fong et al. [22]	Chinese version/18 items	569 Chinese adults	*α* = 0.83, test‐retest = 0.87	AVE = −0.14 and 0.28	*χ* ^2^ = 234.2, CFI = 0.904, RMSEA = 0.055, SRMR = 0.061

Abbreviations: AVE = Convergent validity, CV = Concurrent validity, CR = Composite reliability, FL = Factor loading, *α* = Cronbach's Alpha (internal consistency), *ω* = Omega, CFI, TLI, RMSEA, SRMR = Model fit indices.

Rajshahi ranked fourth in terms of noise pollution, as reported by UNDP, the Ministry of Environment, and many daily newspapers in Bangladesh [30]. Researchers have conducted several studies to measure noise levels in Bangladesh from a geographical and engineering perspective, but hardly any research has taken a psychological perspective. However, it is vital to know people's level of noise sensitivity and determine its effect on mental health.

To date, the NSS scale has not been applied to the Bangladeshi population for evaluating participants' noise sensitivity due to a lack of cross‐cultural adaptation. Specifically, our main purpose of the present study was to assess the psychometric properties of the NSS‐SF‐BV, including its factor structure, reliability, and validity.

## 2 Methods and Materials

### 2.1 Study Design

**Figure 1:**
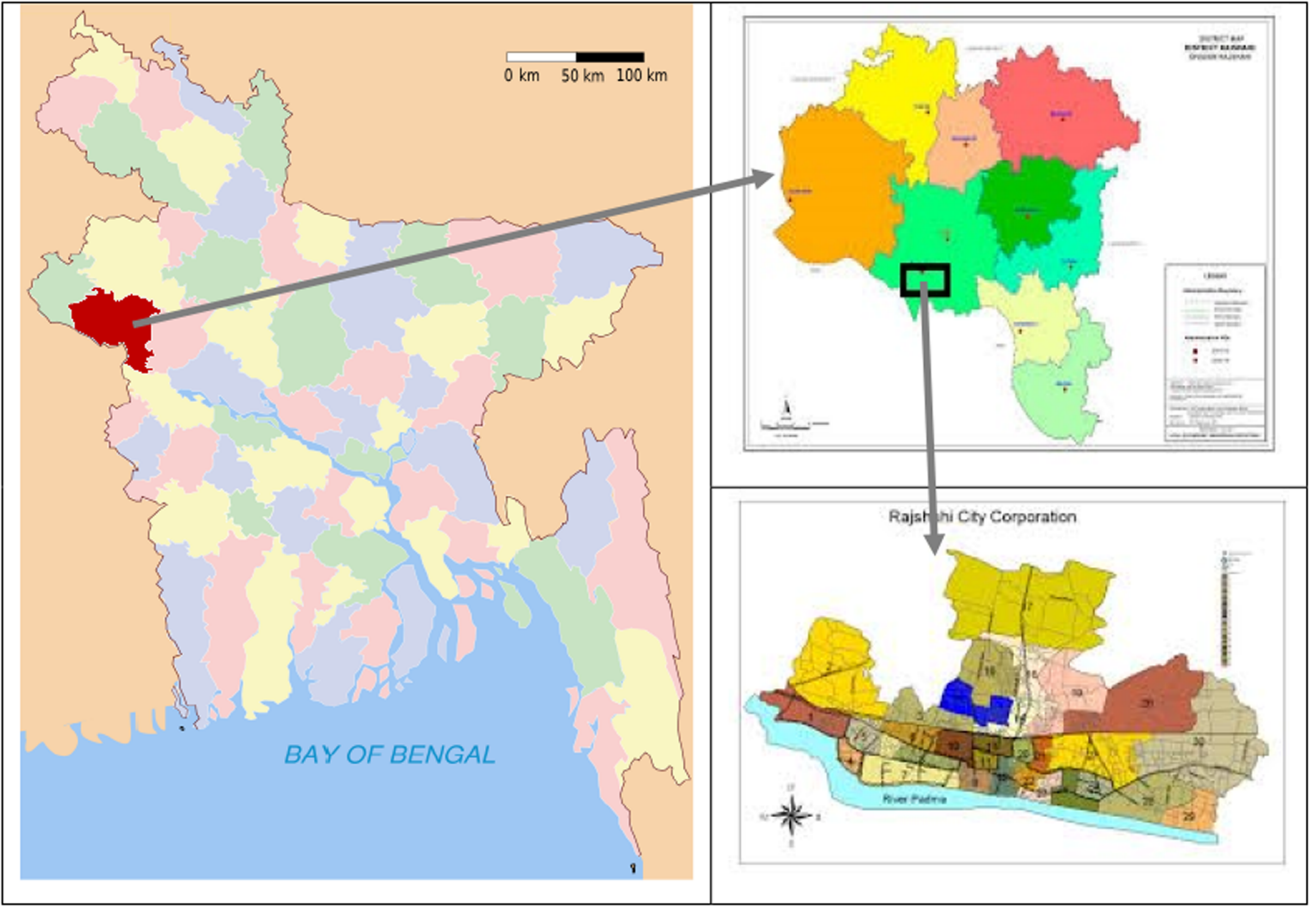
Site map of the study area (Source: Rajshahi district administration).

A cross‐sectional survey was carried out in this study. The authors selected the respondents using convenience sampling techniques. The study was conducted at Rajshahi Metropolitan City, Rajshahi, situated in the northwestern part of Bangladesh. The city is located between 24°20' to 24°24' N latitudes and 88°32' to 88°40' E longitudes, with a total of 95.56 sq km (Figure 1). This area was selected due to its high noise pollution compared to other cities [30]. The data were collected from 10/10/23 to 10/6/24. Student participants were recruited from seven departments across five educational institutions, and general participants were recruited from various places, like shops, homes, streets, markets, and workplaces in the city.

### 2.2 Ethical Approval

This study was approved by the Institutional Animal, Medical Ethics, Biosafety, and Biosecurity Committee (IAMEBBC), Institute of Biological Sciences (IBSc), University of Rajshahi (Reference No: 455(12)/320/IAMEBBC/IBSc; Date: 14/09/2023). The author also followed the ethical principles of the Helsinki Declaration throughout the investigation. Researchers supplied the participants with a brief informed consent document that explicitly articulated all ethical aspects. Confidentiality was upheld, and no personal information, like names, addresses, or contact details, was gathered. Participants did not receive any financial compensation and retained the option to refuse to answer any questions at any time.

### 2.3 Study Participants and Eligibility Criteria

A total of 793 participants with two independent samples (student sample and general sample) were recruited in this study. All respondents were aged between 18 and 69 years old, and Bangla is their language. The participant's eligibility criteria are that all data collected from the Rajshahi City area, and the participant has lived there for less than 1 year.

### 2.4 Sample Size Determination

To explore the sample size for EFA, a total sample of 250–300 is recommended [31]. This study's sample size fulfilled this criterion. To ensure the reliability of the results, this study adhered to the standard practice of using 10 respondents per item for CFA analysis [32, 33, 34, 35]. The Bangla version of NSS‐SF has five items; thus, at least 50 respondents were required. However, this study's sample size exceeded the requirement well.

### 2.5 Instruments

#### 2.5.1 Weinstein Noise Sensitivity Scale Short Form (WNSS‐SF)

The Bangla version of the Weinstein Noise Sensitivity Scale‐short Form (WNSS‐SF‐BV) comprises a 5‐item unidimensional scale [25], which originates from the long‐form 21‐item scale [13]. The five items used in the short form scale were originally coded as items 7, 8, 18, 19, and 21. (Item 7: I get annoyed when my neighbors are noisy; Item 8: I get used to most noises without mass difficulty; Item 18: I find it hard to relax in a place that's noisy; Item 19: I get mad at people who make noise that keeps me from falling asleep or getting work done; and Item 21: I am sensitive to noise.). The six‐point Likert‐type scale ranges from “strongly disagree (1)” to “strongly agree (6).” One of the five items, Item 8, receives a negative code. The final score was obtained by reversing item 8 and then summing all the items. On the present scale, scores range from 6 to 30. A higher score indicates a higher degree of noise sensitivity.

#### 2.5.2 Noise Annoyance Scale (NAS)

The International Commission on the Biological Effects of Noise (ICBEN, ISO/TS‐15666:2021) recommended a standard question for assessing noise annoyance [36]. The Noise Annoyance Scale (NAS) was a five‐point verbal scale of “not annoyed to very high annoyed” as follows: Thinking about the last 12 months, when you are at home or work, how much overall noise bothers, disturbs, or annoys you?

#### 2.5.3 Depression, Anxiety, and Stress Scale (DASS‐21)

The DASS‐21 is a widely used tool to measure the prevalence of depression, anxiety, and stress [37]. The DASS‐21 includes three subscales. Each subscale has 7 items (0 = never to 3 = almost always). This study used the Bengali form of the DASS‐21 [38]. The original Bangla version had Cronbach's alphas of 0.99 for depression, 0.96 for anxiety, and 0.96 for stress subscales. The authors summed the seven items in each subscale and multiplied the result by two to obtain the total score. Each subscale's total score varies from 0 to 42. A higher score indicates a higher severity of depression, anxiety, and stress. Ahmed et al. [39] reported that the DASS‐21 demonstrates excellent psychometric properties.

### 2.6 Translation, Back Translation, Pilot Testing, and Procedure

This study followed the guidelines of the International Test Commission (2018) [40] for translating and adapting the NSS‐SF‐BV items to ensure equivalency. At first, two English literature professors independently translated the WNSS‐SF items into Bangla. A comparison was made between both professors ‍and back‐translated into English by two professors of the Bangla language. In the same way, a comparison was made between both professors, modified until a compromise was achieved between the Bangla and English versions. Two psychology professors were then allowed to test the scale. Minor revisions are made based on their feedback. All six professors had comprehensive knowledge of the cultural context. Then, a focus group discussion (FGD) was conducted with eight master's students who have a proficient command of both languages in the Department of Clinical Psychology, University of Rajshahi, to determine the people's language equivalence and understandability. Their suggestions were accepted. A pilot study with a pre‐final version was conducted with a total of 36 student participants.

The person‐to‐person, structured, self‐reported, and anonymous survey questionnaire was employed which consisted of a cover page including informed consent forms such as the purpose of the research, a second‐page containing demographic variables and behavioral information (i.e., age and gender), and the remaining page containing four psychological scales, e.g., the NSS, NAS, and DASS‐21. The authors collected the data using a trained data collector with a background in psychology. For test‐retest reliability, data were collected at 2 to 3 weeks intervals.

### 2.7 Statistical Analysis

SPSS v26, SPSS AMOS v24 and Microsoft Excel 2019 were used for data management and analysis. Descriptive statistics, such as mean, standard deviation, skewness, kurtosis, and intercorrelation among the items of the NSS‐SF‐BV were calculated. Skewness, kurtosis, and the Kolmogorov‐Smirnov test were used to assess the data's normality. According to Kline, skewness < 2 and kurtosis < 4 indicate the data normality of the large sample [41].

Reliability (Cronbach's alpha and Omega), convergent validity (AVE‐average variance extracted), composite reliability (CR) and the corrected item‐total correlation (*r*_tt_) were utilized for internal consistency and accepted a cutoff score above or equal to 0.70 [42, 43, 44, 45, 46]. In the case of corrected item‐total score correlations (*r*_tt_), Hoe [42] and Field [46] recommended that a cutoff score above 0.3 be applied (Table 3).

Composite reliability (CR) and convergent validity (AVE) were calculated from factor loading through Microsoft Excel 2019. The formula of the composite reliability, CR = (∑ *λ*)^2^/(∑ *λ*)^2^ + ∑*ε*); and the formula of the average variance extracted, AVE = ∑*λ* ^2^/*N*; here, *λ* = factor loading, *ε* = 1–*λ* ^2^, *N* = numbers of items, but correlations of the NSS‐SF‐BV with the DASS‐21 subscales and NAS were established to assess the calculate concurrent validity of the NSS‐SF‐BV.

The EFA was conducted to assess the factor structure of the NSS‐SF‐BV. The Kaiser‐Meyer‐Olkin (KMO) > 0.60 [47]; and Barlett's test of sphericity (*p* < 0.001) [48], test was applied to ensure that the data set is suitable or not for EFA. For both EFA and CFA, a cutoff score above 0.3 was applied for factor loading [42, 46].

A CFA was conducted with the maximum likelihood technique to assess the factorial validity of NSS‐SF‐BV. According to Hu and Bentler [49], the model fits indices including the nonsignificant Chi‐square value (*χ* ^2^), the comparative fit index (CFI) ≥ 0.90, the Tucker–Lewis index (TLI) ≥ 0.90, the goodness of fit index (GFI) ≥ 0.90, and the root‐mean‐square error of approximation (RMSEA) ≤ 0.08, were estimated to ensure the suitability of the model (refer to Table 5 for the cutoff scores). EFA and CFA were calculated using SPSS version 26 and SPSS AMOS version 24, respectively.

## 3 Results

### 3.1 Demographic Characteristics of the Participants

After excluding 53 participants due to outliers and Mahalanobis distance checks, A total of 793 participants were confirmed in this study (mean_age_ = 28.23, SD_age_ = 9.42, range_age_ = 18–69), of which 357 (45%) are males and 436 (55%) are females. Among them, 491 participants were college and university students (mean_age_ = 22.90, SD_age_ = 2.04, range_age_ = 18–28), and 302 participants were general people (mean_age_ = 36.90, SD_age_ = 10.25, range_age_ = 19–69). The student sample comprised 205 males (41.8%) and 286 females (58.2%). On the other hand, the general sample comprised 152 males (50.3%) and 150 females (49.7%) (Table 2).

**Table 2:** Participants' characteristics.

Variable	Student sample (*n*_1_ = 491)	General sample (*n*_2_ = 302)	Total sample (*n* = 793)
Age (years)			
Mean ± SD	22.90 ± 2.04	36.90 ± 10.25	28.23 ± 9.42
Range	18–28	19–69	18–69
Gender, *n* (%)			
Male	205 (41.8%)	152 (50.3%)	357 (45.02%)
Female	286 (58.2%)	150 (49.7%)	436 (54.98%)

### 3.2 Descriptive Statistics of Each Item of NSS‐SF‐BV

The mean, standard deviation (SD), skewness, and kurtosis of each item of the NSS‐SF‐BV scale were calculated (Table 3). The means and standard deviations of the items were 4.14–5.20 and 0.79–1.14, respectively. Furthermore, the skewness and kurtosis values (e.g., −0.01 to −1.18 and −0.06 to 1.48, respectively) were found within an acceptable range (e.g., skewness < 2 and kurtosis < 4) [34]. These results supported that the data set did not violate normality. The corrected item‐total correlation (*r*_tt_) indicated a coefficient above 0.30 (0.345–0.642). In the case of corrected item‐total score correlations (*r*_tt_), a cutoff score above 0.3 is applied [42, 48]. (Table 3).

**Table 3:** Means, standard deviations (SD), skewness, kurtosis, and item‐total score corrected correlations (*r*_tt_) of each item of the NSS‐SF‐BV scale for two samples.

	Student sample (*n*_1_ = 491)					General sample (*n*_2_ = 302)				
Items	Mean	*SD*	Sk	Ku	*r*_tt_	Mean	*SD*	Sk	Ku	*r*_tt_
Item 7	4.91	0.80	−0.55	0.17	0.56	4.84	0.99	−0.87	0.48	0.45
Item 8	4.16	0.88	−0.19	−0.54	0.35	4.21	0.76	−0.01	0.09	0.56
Item 18	5.20	0.80	−0.90	0.50	0.58	5.09	0.92	−1.18	1.48	0.61
Item 19	5.16	0.79	−0.69	−0.06	0.64	5.14	0.85	−1.06	1.09	0.48
Item 21	4.60	0.90	−0.39	−0.22	0.53	4.46	1.14	−0.32	−0.75	0.42

Abbreviations: Ku = Kurtosis, *r*_tt =_ item‐total score corrected correlations, Sk = Skewness.

### 3.3 Exploratory Factor Analysis (EFA) of NSS‐SF‐BV

**Figure 2:**
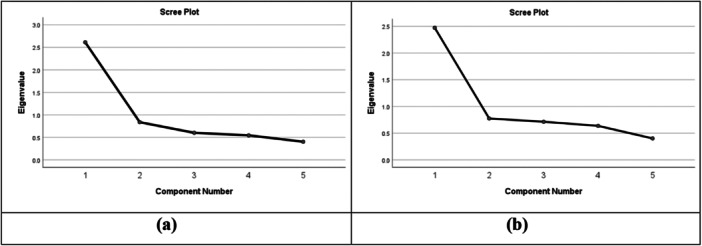
Scree plot of two samples, (a) student sample and (b) general sample.

For the EFA (student sample), Kaiser‐Meyer‐Olkin (KMO) was found 0.805 ( > 0.60) [47]; and Barlett's test of sphericity was significant (*χ* ^2^ = 603.52, *df* = 10, *p*‐value < 0.001) [48], among the NSS‐SF‐BV, which indicated that the data set was suitable, and adequate correlation matrix for factor analysis. The value of inter‐item correlation coefficients ranged between 0.281 and 0.560, suggesting within the acceptable range (Table 3). The EFA results confirmed the NSS‐SF‐BV's one‐factor structure with five items. The results revealed one factor, which explained 52.23% of the total variance. The scree plot also showed a clear discontinuity after the first factor. The results were consistent with previous results [6, 23, 27]. (Figure 2).

### 3.4 Confirmatory Factor Analysis (CFA) of NSS‐SF‐BV

The CFA results revealed that the one‐factor unidimensional model recognized for the NSS‐SF‐BV. For the student sample, model fit indices showed a good fit: *χ* ^2^ = 6.21, *df* = 5, *p*‐value = 0.286, *χ* ^2^/*df* ratio = 1.24, CFI = 0.998, TLI = 0.996, and RMSEA = 0.022. In terms of the general sample, all results also indicate the model fits perfectly except p‐value: *χ* ^2^ = 13.87, *df* = 5, *p*‐value = 0.016, *χ* ^2^/*df* ratio = 2.77, CFI = 0.971, TLI = 0.942, and RMSEA = 0.077. However, Kline (2015) [34] suggests that the chi‐square test is highly sensitive to sample size, meaning that a minor deviation alone does not necessarily indicate a poor fit (Table 5). However, these findings indicate that the NSS‐SF‐BV have structural validity across various groups. The factor loading (standardized regression weights) of each item ranged from 0.375 and 0.793 for the student sample and 0.455– 0.770 for the general sample (Table 4).

**Table 4:** Factor loading of the unidimensional structure obtained from CFA.

Item no and content *(according to the original scale)*	Factor loading (student sample, *n*_1_ = 491)	Factor loading (general sample, *n*_2_ = 302)
Item 7: I get annoyed when my neighbors are noisy.	0.657	0.520
Item 8: I get used to most noises without mass difficulty.	0.367	0.455
Item 18: I find it hard to relax in a place that's noisy.	0.715	0.708
Item 19: I get mad at people who make noise that keeps me from falling asleep or getting work done.	0.793	0.770
Item 21: I am sensitive to noise.	0.610	0.561

### 3.5 Internal Consistency of the NSS‐SF‐BV

Cronbach's alpha values were found 0.76 for the student sample, 0.75 for the general sample and 0.75 for the combined sample and Omega values were found 0.79 for the student sample, 0.79 for the general sample and 0.78 for the combined sample, respectively, indicating an acceptable internal consistency [50] (Table 5) The item‐total score corrected correlations (Rtt) were also found in the acceptable range (range from 0.35 to 0.64 for the student sample and 0.42–0.61 for the general sample). According to Hoe [42] and Field [46], the accepted level of corrected item‐total score correlations (rtt) is above 0.3 (Table 3). The previous results clearly stated that the NSS‐SF‐BV had acceptable reliability [50]. To examine test‐retest reliability, the Pearson Correlation between the test and retest total score and the correlation was found 0.72 (*p* < 0.001, *N* = 81) over a 2‐week interval (Table 5) [50]. Additionally, composite reliability (CR) was found to be 0.77 for the student sample and 0.76 for the general sample, indicating an acceptable composite reliability [45] (Table 5).

**Table 5:** Psychometric properties of the NSS‐SF‐BV.

Psychometric properties	Student sample (*n*_1_ = 491)	General sample (*n*_2_ = 302)	Cutoff score
Cronbach's alpha	0.76	0.75	≥ 0.70 Wuang et al. [50]
McDonald's omega	0.79	0.79	≥ 0.70 Wuang et al. [50]
Test‐retest reliability	0.72 (*n* = 81)	—	≥ 0.70 Wuang et al. [50]
Composite reliability	0.77	0.76	≥ 0.70 Fornell & Larcker [45]
AVE	0.86	0.71	≥ 0.70 Wuang et al. [50]
Model fits of CFA			
*χ* ^2^ (*df*, *p*‐value)	6.21 (5, 0.286)	13.87 (5, 0.016)	Nonsignificant Hu & Bentler [49]
*χ* ^2^/*df*	1.24	2.77	< 3 Kline [41], Hoe [42]
CFI	0.998	0.971	≥ 0.95 Hu & Bentler [49]
TLI	0.996	0.942	≥ 0.95 Hu & Bentler [49]
RMSEA	0.022	0.077	< 0.08 Hu & Bentler [49]

### 3.6 Validity of the NSS‐SF‐BV

To estimate concurrent validity, the noise annoyance scale and DASS‐21 subscales were used. Researchers administered the Pearson correlation among the NSS‐SF‐BV, NAS, and DASS‐21 subscales for the purpose (Table 6). From the calculations, a positive and strong association was found with NAS (*r* = 0.381, *p* = 0.01), a significant but weak correlation with stress (*r* = 0.089, *p* = 0.05), and a nonsignificant correlation with depression (*r* = 0.025) and anxiety (*r* = 0.023) scales (Table 6). Note that other studies [15, 17] used trait anxiety scales to examine concurrent validity. However, due to the lack of a trait anxiety scale in the Bengali version, researchers have utilized the DASS‐21 and NAS scales. No previous studies have used the DASS‐21 scales to examine the concurrent validity of the NSS; instead, trait anxiety [23] and stress scales were used. This likely explains why this study did not find any correlation between the NSS and the depression as well as anxiety subscales. To estimate convergent validity (AVE), this study considered the factor loading of the items and composite validity (CR). Convergent validity (AVE) was found 0.86 for the student sample and 0.71 for the general sample, representing the convergent validity of the NSS‐SF‐BV [50] (Table 5).

**Table 6:** Pearson correlation of the Bangla version of NSS‐SF with NAS and DASS‐21 subscales.

Others scale	NSS‐SF
NAS	0.46**
Depression subscale	0.03
Anxiety subscale	0.02
Stress subscale	0.09*

** Correlation is significant at the 0.01 level (two‐tailed).

* Correlation is significant at the 0.05 level (two‐tailed).

## 4 Discussion

Understanding the impact of noise on mental health is crucial. People who have a high sensitivity to noise tend to experience more irritation. Those with high noise sensitivity experience a decrease in their health‐related quality of life, an increase in heart disease risk, and a decrease in sleep quality, among other negative effects. The main purpose of the NSS/NSS‐SF scale is to assess individual differences in noise sensitivity. As noted in the introductory chapter, this scale is widely accepted and usable. The original NSS scale had 21 items, but the current adapted NSS‐SF‐BV scale has only 5. This NSS‐SF simplified the field study, facilitated the collection of more data in a shorter time, reduced the participant burden, and ensured the availability of reliable data [25]. However, this also maintains the original scale's level of scientific rigor.

However, the present study aimed to assess the NSS‐SF‐BV in the context of Bangladesh, as well as determine its factor structure, reliability, and validity for nonclinical settings. To assess the psychometric properties of the NSS‐SF‐BV, descriptive statistics, data normality, Cronbach's alpha, McDonald omega, test‐retest reliability, composite reliability, convergent validity, concurrent validity, EFA and CFA were tested.

The present study consisted of two separate samples: the student sample (*n*_1_ = 491) and the general population sample (*n*_2_ = 302), for a combined sample size of 793 participants. The student sample consisted of young adults aged between 18 and 28 years. Additionally, the original scale was developed in a longitudinal study on a student sample [13]. The general population sample consisted of young adults, aged between 19 and 69 years. The general population sample included a diverse range of ages, employment, educational and socioeconomic status.

Table 2 shows the means, standard deviations, skewness, kurtosis, and item‐total score corrected correlations (*r*_tt_) of each item of the NSS‐SF‐BV. Both samples indicated that all items confirmed variability, skewness, and kurtosis within an acceptable range. The item‐total score corrected correlation values show above the cutoff values, which reflects internal consistency and contribution of each item to the total score. The mean values of both samples suggest that this scale can perform consistently across various ages and backgrounds. These results are consistent with previous validation studies [17, 18, 20, 21, 22, 23, 24, 25, 26, 27].

An EFA was demonstrated to examine the underlying structure of the NSS‐SF‐BV. The KMO was found to be 0.805, suggesting that the data were well‐suited for factor analysis [47]. Bartlett's test of sphericity was significant, indicating adequate correlation between the items to justify EFA. The inter‐item correlation coefficients were found acceptable range, the items are correlated but not redundant. The EFA showed a one‐factor solution, explaining 52.23% of total variance. This result showed that all five items load significantly on a single factor. These findings are consistent with prior studies [22, 51]. The scree plot also confirmed a specific breakdown after the first factor, suggesting the unidimensional features of the scale [18, 20, 25].

The CFA was administered to assess the factorial structure of the NSS‐SF‐BV. The model fit indices suggested a good model fit. The chi‐square and chi‐square/df values were shown within the recommended range, indicating a good model fit. The CFI and TLI exceeded the recommended threshold, suggesting a good model fit. RMSEA were also within the recommended threshold, indicating adequacy of the model. Additionally, all items of the NSS‐SF‐BV exhibited acceptable factor loadings, indicating that each item represents the latent construct of noise sensitivity [20, 23, 25, 26, 27].

The NSS‐SF‐BV demonstrated good internal consistency across different indices. The Cronbach's alpha was above the cutoff value, indicating good internal consistency and suggesting that the scale items consistently measure the same underlying construct [20]. Moreover, McDonald's Omega was above the recommended value and further confirms the internal consistency [17, 18, 20, 21, 22, 23, 24, 25, 26, 27].

To estimate the temporal stability of the NSS‐SF‐BV, a test‐retest reliability was conducted over a 2‐week time interval, indicating good stability of the scale. Additionally, the scale showed adequate composite reliability, which exceeded the recommended value. These results revealed that the scale has good internal consistency and is appropriate for the Bangladeshi population [18, 20, 21, 22, 24].

The validity of the NSS‐SF‐BV was assessed through two approaches, including concurrent validity and convergent validity. To estimate concurrent validity, the Pearson correlation coefficients were administered between the NAS, depression, anxiety and stress scale. The results indicated the NSS‐SF‐BV scores were positively and significantly associated with NAS [3, 52, 53, 54] and stress scores [12, 55, 56, 57, 58, 59, 60], which suggests concurrent validity. Additionally, the convergent value was found above the recommended value. These findings are consistent with previous studies, demonstrating that people with higher noise sensitivity tend to experience greater noise annoyance and mental health distress [23, 25, 26].

In conclusion, the present research provides strong evidence for the psychometric properties of NSS‐SF‐BV. The scale showed good internal consistency, satisfactory test‐retest reliability, acceptable validity and a unidimensional factor structure across both samples. These findings suggest that the NSs‐SF‐BV is a valid and reliable tool for assessing noise sensitivity among Bangladeshi people.

### 4.1 Limitations

One of the study's major limitations is the use of a nonrandom sampling technique in selecting the respondents. All data were gathered from Rajshahi City; it would have been more beneficial to gather information from people from various locations, like rural areas and other cities, for comparing the results. Moreover, participants' responses varied due to the influence of culture and the rapidly changing generation. A few studies have reported the need for two‐item modifications (original scale items 7 and 18), and many have referred to the 6‐point Likert‐type scale as a response bias [21, 26]. Another observation in an underdeveloped country is that most people are unwilling to accept noise pollution, likely due to their ignorance about pollution and the potential harm it can cause to their physical and mental health.

### 4.2 Implications and Future Directions

Despite its limitations, the psychometric properties of this scale are quite good. Because of the lack of a Bangla‐adapted NSS tool, this NSS‐SF‐BV scale will be an effective tool for assessing people's noise sensitivity. This tool may perform a significant role in psychological assessment for determining the level of noise sensitivity and how noise sensitivity affects people's mental health in Bangladesh, and it may also be convenient for research regarding noise sensitivity.

## 5 Conclusion

This study has revealed that the NSS‐SF‐BV is a unidimensional, personal‐centered, and psychometrically sound tool for assessing the noise sensitivity of Bangladeshi individuals. The CFA confirmed the factor structure. Given the prevalence of noise pollution in Bangladesh, this tool will support concerned researchers in conducting a field‐based study to understand the detrimental effects of noise sensitivity. Urban planners can implement appropriate intervention strategies to control noise pollution and reduce its negative effects on the general public.

## Author Contributions

**Pramath Chandra Sarker:** conceptualization, investigation, writing – original draft, methodology, validation, visualization, writing – review and editing, software, formal analysis, data curation, resources. **Md Nur‐E‐Alam Siddique:** conceptualization, writing – review and editing, data curation, methodology, supervision, project administration. **Sabina Sultana:** supervision, Data curation, project administration, conceptualization.

## Disclosure

The lead author Pramath Chandra Sarker affirms that this manuscript is an honest, accurate, and transparent account of the study being reported; that no important aspects of the study have been omitted; and that any discrepancies from the study as planned (and, if relevant, registered) have been explained.

## Ethics Statement

Before questionnaire administration, ethical clearance has been obtained from the *Institutional Animal, Medical Ethics, Biosafety and Biosecurity Committee (IAMEBBC)* for Experimentations on Animal, Human, Microbes and Living Natural Sources at the Institute of Biological Sciences (IBSc), University of Rajshahi Research Ethics Committee (Reference No.: 455(12)/320/IAMEBBC/IBSc; Date: 14/09/2023). Ethical clearance has been obtained as part of the original PhD research (the same survey and questionnaire), and this article is carried out as part of the partial fulfillment of the PhD requirements.

## Conflicts of Interest

The authors declare no conflicts of interest.

### Transparency Statement

The three authors collected the data, and the lead author, Pramath Chandra Sarkar, did the formal analysis and original draft writings. The three authors read and confirm that this manuscript is honest, accurate, and transparent. The authors also ensured that this study did not omit any important aspects and that any discrepancies from the study were explained as planned.

## Data Availability

The data that support the findings of this study are available from the corresponding author upon reasonable request.
